# 7*H*-Chromeno[3,2-*h*]quinolin-7-one methanol monosolvate

**DOI:** 10.1107/S160053680903534X

**Published:** 2009-09-09

**Authors:** Jiang-Ke Qin, Zheng-Min Yang, Ming-Hua Zeng, Seik Weng Ng

**Affiliations:** a Key Laboratory for the Chemistry & Molecular Engineering of Medicinal Resources, (Ministry of Education of China), School of Chemistry & Chemical Engineering, Guangxi Normal University, 541004 Guilin 541004, People’s Republic of China; b Department of Chemistry, University of Malaya, 50603 Kuala Lumpur, Malaysia

## Abstract

The four-ring system in the title compound, C_16_H_9_NO_2_·CH_3_OH, is planar (r.m.s deviation = 0.03 Å); the methanol solvent mol­ecule forms a hydrogen bond to the quinoline N atom.

## Related literature

The compound in this study was synthesized from the cycliz­ation of 2-(quinolin-8-yl­oxy)benzoic acid; for the synthesis of this acid, see: Chen *et al.* (2007 ▶). For the synthesis by the Skraup reaction of amino-9*H*-xanthene-9-one, see: Fujiwara & Okabayashi (1994 ▶).
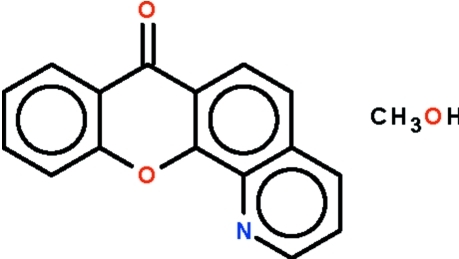


## Experimental

### 

#### Crystal data

C_16_H_9_NO_2_·CH_4_O
*M* *_r_* = 279.28
Triclinic, 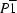

*a* = 8.102 (2) Å
*b* = 8.791 (3) Å
*c* = 10.150 (3) Å
α = 102.172 (3)°
β = 108.760 (3)°
γ = 93.532 (3)°
*V* = 662.6 (3) Å^3^
*Z* = 2
Mo *K*α radiation
μ = 0.10 mm^−1^
*T* = 295 K
0.41 × 0.30 × 0.18 mm

#### Data collection

Bruker APEXII diffractometer
Absorption correction: none
3838 measured reflections
2655 independent reflections
1456 reflections with *I* > 2σ(*I*)
*R*_int_ = 0.016

#### Refinement

*R*[*F* ^2^ > 2σ(*F* ^2^)] = 0.046
*wR*(*F* ^2^) = 0.147
*S* = 1.02
2655 reflections
192 parameters
H-atom parameters constrained
Δρ_max_ = 0.22 e Å^−3^
Δρ_min_ = −0.15 e Å^−3^

### 

Data collection: *APEX2* (Bruker, 2004 ▶); cell refinement: *SAINT* (Bruker, 2004 ▶); data reduction: *SAINT* ▶); program(s) used to solve structure: *SHELXS97* (Sheldrick, 2008 ▶); program(s) used to refine structure: *SHELXL97* (Sheldrick, 2008 ▶); molecular graphics: *X-SEED* (Barbour, 2001 ▶); software used to prepare material for publication: *publCIF* (Westrip, 2009 ▶).

## Supplementary Material

Crystal structure: contains datablocks I, global. DOI: 10.1107/S160053680903534X/xu2605sup1.cif

Structure factors: contains datablocks I. DOI: 10.1107/S160053680903534X/xu2605Isup2.hkl

Additional supplementary materials:  crystallographic information; 3D view; checkCIF report

## Figures and Tables

**Table 1:** Hydrogen-bond geometry (Å, °)

*D*—H⋯*A*	*D*—H	H⋯*A*	*D*⋯*A*	*D*—H⋯*A*
O3—H3⋯N1	0.82	2.07	2.852 (2)	160

**Fig. 1.**
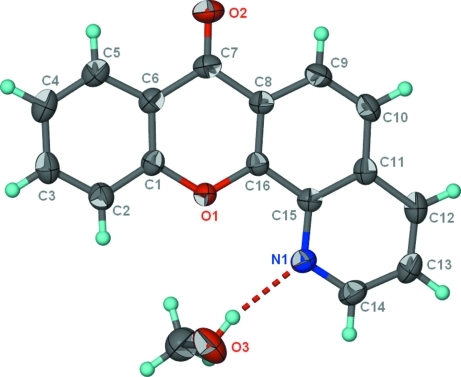
Thermal ellipsoid plot (Barbour, 2001) of C16H9NO2.CH4O at the 50% probability level; hydrogen atoms are drawn as sphere of arbitrary radius. The dashed line denotes a hydrogen bond.

## supplementary crystallographic information

### Experimental

2-(Quinolin-8-yloxy)benzoic acid was synthesized by using a literature procedure (Chen *et al.*, 2007). The carboxylic acid (0.5 g) and polyphosphoric acid (3.5 g) were heated at 413 K for two hours; the reaction was monitored by thin layer chromatography. The hot mixture was poured into ice water (200 ml); the pH value of the solution was adjusted to 7–8 by concentrated ammonium hydroxide. The crude product that precipitated was collected and recrystallized from methanol (in 0.39 g yield). The formulation was established by ^1^H NMR spectral integral analysis.

### Refinement

Carbon- and oxygen-bound hydrogen atoms were generated geometrically and were constrained to ride on their parent atoms [C–H = 0.93–0.96, O–H 0.82 Å; *U*_iso_(H) =1.2–1.5*U*_eq_(C,O)].

### Crystal data

**Table:**

C_16_H_9_NO_2_·CH_4_O	*Z* = 2
*M**_r_* = 279.28	*F*(000) = 292
Triclinic, *P*1	*D*_x_ = 1.400 Mg m^−^^3^
Hall symbol: -P 1	Mo *K*α radiation, λ = 0.71073 Å
*a* = 8.102 (2) Å	Cell parameters from 1000 reflections
*b* = 8.791 (3) Å	θ = 2.2–26.5°
*c* = 10.150 (3) Å	µ = 0.10 mm^−^^1^
α = 102.172 (3)°	*T* = 295 K
β = 108.760 (3)°	Block, yellow
γ = 93.532 (3)°	0.41 × 0.30 × 0.18 mm
*V* = 662.6 (3) Å^3^	

### Data collection

**Table:**

Bruker APEXII diffractometer	1456 reflections with *I* > 2σ(*I*)
Radiation source: fine-focus sealed tube	*R*_int_ = 0.016
graphite	θ_max_ = 26.5°, θ_min_ = 2.2°
φ and ω scans	*h* = −10→10
3838 measured reflections	*k* = −9→11
2655 independent reflections	*l* = −12→12

### Refinement

**Table:**

Refinement on *F*^2^	Primary atom site location: structure-invariant direct methods
Least-squares matrix: full	Secondary atom site location: difference Fourier map
*R*[*F*^2^ > 2σ(*F*^2^)] = 0.046	Hydrogen site location: inferred from neighbouring sites
*wR*(*F*^2^) = 0.147	H-atom parameters constrained
*S* = 1.02	*w* = 1/[σ^2^(*F*_o_^2^) + (0.0688*P*)^2^ + 0.0458*P*] where *P* = (*F*_o_^2^ + 2*F*_c_^2^)/3
2655 reflections	(Δ/σ)_max_ = 0.001
192 parameters	Δρ_max_ = 0.22 e Å^−^^3^
0 restraints	Δρ_min_ = −0.15 e Å^−^^3^

### Fractional atomic coordinates and isotropic or equivalent isotropic displacement parameters (Å^2^)

**Table:**

	*x*	*y*	*z*	*U*_iso_*/*U*_eq_	
O1	0.25297 (18)	0.41753 (15)	0.57244 (14)	0.0387 (4)	
O2	0.2583 (2)	0.64419 (19)	0.25985 (17)	0.0601 (5)	
O3	0.2226 (2)	0.2382 (2)	0.8300 (2)	0.0735 (6)	
H3	0.1946	0.3170	0.8021	0.110*	
N1	0.1800 (2)	0.5518 (2)	0.80538 (18)	0.0402 (5)	
C1	0.2905 (3)	0.3418 (2)	0.4545 (2)	0.0359 (5)	
C2	0.3238 (3)	0.1887 (3)	0.4504 (2)	0.0449 (6)	
H2	0.3204	0.1427	0.5242	0.054*	
C3	0.3619 (3)	0.1058 (3)	0.3359 (3)	0.0506 (6)	
H3a	0.3839	0.0027	0.3318	0.061*	
C4	0.3680 (3)	0.1741 (3)	0.2259 (3)	0.0535 (6)	
H4	0.3943	0.1169	0.1489	0.064*	
C5	0.3353 (3)	0.3254 (3)	0.2310 (2)	0.0475 (6)	
H5	0.3398	0.3706	0.1570	0.057*	
C6	0.2950 (3)	0.4136 (2)	0.3461 (2)	0.0374 (5)	
C7	0.2553 (3)	0.5754 (2)	0.3526 (2)	0.0401 (5)	
C8	0.2134 (2)	0.6488 (2)	0.4794 (2)	0.0341 (5)	
C9	0.1720 (3)	0.8044 (2)	0.4991 (2)	0.0429 (6)	
H9	0.1653	0.8579	0.4280	0.052*	
C10	0.1423 (3)	0.8764 (3)	0.6186 (3)	0.0445 (6)	
H10	0.1182	0.9795	0.6301	0.053*	
C11	0.1473 (3)	0.7963 (2)	0.7271 (2)	0.0372 (5)	
C12	0.1185 (3)	0.8653 (3)	0.8556 (3)	0.0487 (6)	
H12	0.0984	0.9694	0.8738	0.058*	
C13	0.1204 (3)	0.7792 (3)	0.9517 (3)	0.0521 (6)	
H13	0.1023	0.8237	1.0367	0.063*	
C14	0.1498 (3)	0.6225 (3)	0.9221 (2)	0.0476 (6)	
H14	0.1481	0.5646	0.9886	0.057*	
C15	0.1802 (2)	0.6383 (2)	0.7082 (2)	0.0341 (5)	
C16	0.2159 (3)	0.5678 (2)	0.5817 (2)	0.0326 (5)	
C17	0.4047 (4)	0.2427 (3)	0.8712 (3)	0.0733 (8)	
H17A	0.4457	0.2784	0.8026	0.110*	
H17B	0.4345	0.1394	0.8754	0.110*	
H17C	0.4594	0.3135	0.9641	0.110*	

### Atomic displacement parameters (Å^2^)

**Table:**

	*U*^11^	*U*^22^	*U*^33^	*U*^12^	*U*^13^	*U*^23^
O1	0.0529 (10)	0.0317 (8)	0.0361 (9)	0.0084 (7)	0.0207 (7)	0.0087 (6)
O2	0.0888 (13)	0.0568 (11)	0.0475 (10)	0.0125 (9)	0.0329 (10)	0.0240 (8)
O3	0.0733 (14)	0.0473 (11)	0.1034 (17)	0.0123 (9)	0.0283 (12)	0.0276 (10)
N1	0.0469 (11)	0.0408 (11)	0.0351 (11)	0.0057 (8)	0.0163 (9)	0.0102 (8)
C1	0.0344 (12)	0.0363 (12)	0.0334 (12)	0.0019 (9)	0.0112 (10)	0.0025 (9)
C2	0.0480 (14)	0.0386 (13)	0.0490 (14)	0.0068 (10)	0.0187 (12)	0.0094 (11)
C3	0.0538 (15)	0.0383 (13)	0.0572 (16)	0.0093 (11)	0.0207 (13)	0.0025 (11)
C4	0.0523 (16)	0.0563 (16)	0.0487 (16)	0.0073 (12)	0.0223 (13)	−0.0019 (12)
C5	0.0496 (15)	0.0523 (15)	0.0401 (14)	0.0034 (11)	0.0183 (11)	0.0067 (11)
C6	0.0344 (12)	0.0396 (12)	0.0352 (13)	−0.0005 (9)	0.0116 (10)	0.0048 (10)
C7	0.0407 (13)	0.0429 (13)	0.0345 (13)	−0.0020 (10)	0.0108 (10)	0.0103 (10)
C8	0.0324 (12)	0.0331 (11)	0.0343 (12)	−0.0001 (9)	0.0092 (10)	0.0077 (9)
C9	0.0492 (14)	0.0376 (12)	0.0457 (14)	0.0065 (10)	0.0169 (11)	0.0168 (10)
C10	0.0478 (14)	0.0315 (12)	0.0561 (15)	0.0088 (10)	0.0190 (12)	0.0119 (11)
C11	0.0330 (12)	0.0337 (12)	0.0412 (13)	0.0023 (9)	0.0113 (10)	0.0039 (9)
C12	0.0506 (15)	0.0396 (13)	0.0528 (15)	0.0104 (11)	0.0195 (12)	0.0009 (11)
C13	0.0600 (16)	0.0543 (15)	0.0422 (14)	0.0091 (12)	0.0243 (13)	0.0010 (12)
C14	0.0549 (15)	0.0543 (15)	0.0368 (13)	0.0071 (12)	0.0193 (12)	0.0122 (11)
C15	0.0305 (12)	0.0351 (12)	0.0342 (12)	0.0012 (9)	0.0087 (9)	0.0081 (9)
C16	0.0323 (12)	0.0285 (11)	0.0349 (12)	0.0017 (9)	0.0105 (9)	0.0056 (9)
C17	0.077 (2)	0.080 (2)	0.069 (2)	0.0168 (16)	0.0302 (17)	0.0195 (16)

### Geometric parameters (Å, °)

**Table:**

O1—C16	1.363 (2)	C7—C8	1.466 (3)
O1—C1	1.375 (2)	C8—C16	1.373 (3)
O2—C7	1.227 (2)	C8—C9	1.417 (3)
O3—C17	1.394 (3)	C9—C10	1.347 (3)
O3—H3	0.8200	C9—H9	0.9300
N1—C14	1.321 (3)	C10—C11	1.419 (3)
N1—C15	1.367 (2)	C10—H10	0.9300
C1—C2	1.384 (3)	C11—C15	1.415 (3)
C1—C6	1.388 (3)	C11—C12	1.415 (3)
C2—C3	1.371 (3)	C12—C13	1.352 (3)
C2—H2	0.9300	C12—H12	0.9300
C3—C4	1.386 (3)	C13—C14	1.399 (3)
C3—H3a	0.9300	C13—H13	0.9300
C4—C5	1.366 (3)	C14—H14	0.9300
C4—H4	0.9300	C15—C16	1.429 (3)
C5—C6	1.403 (3)	C17—H17A	0.9600
C5—H5	0.9300	C17—H17B	0.9600
C6—C7	1.470 (3)	C17—H17C	0.9600
			
C16—O1—C1	118.65 (15)	C8—C9—H9	119.3
C17—O3—H3	109.5	C9—C10—C11	120.6 (2)
C14—N1—C15	117.14 (18)	C9—C10—H10	119.7
O1—C1—C2	115.76 (18)	C11—C10—H10	119.7
O1—C1—C6	122.41 (18)	C15—C11—C12	116.89 (19)
C2—C1—C6	121.82 (19)	C15—C11—C10	119.7 (2)
C3—C2—C1	119.0 (2)	C12—C11—C10	123.4 (2)
C3—C2—H2	120.5	C13—C12—C11	119.7 (2)
C1—C2—H2	120.5	C13—C12—H12	120.2
C2—C3—C4	120.7 (2)	C11—C12—H12	120.2
C2—C3—H3a	119.6	C12—C13—C14	119.4 (2)
C4—C3—H3a	119.6	C12—C13—H13	120.3
C5—C4—C3	119.9 (2)	C14—C13—H13	120.3
C5—C4—H4	120.0	N1—C14—C13	123.9 (2)
C3—C4—H4	120.0	N1—C14—H14	118.0
C4—C5—C6	121.0 (2)	C13—C14—H14	118.0
C4—C5—H5	119.5	N1—C15—C11	122.95 (19)
C6—C5—H5	119.5	N1—C15—C16	119.13 (18)
C1—C6—C5	117.6 (2)	C11—C15—C16	117.92 (18)
C1—C6—C7	120.34 (19)	O1—C16—C8	123.74 (18)
C5—C6—C7	122.08 (19)	O1—C16—C15	115.04 (16)
O2—C7—C8	122.5 (2)	C8—C16—C15	121.22 (18)
O2—C7—C6	122.8 (2)	O3—C17—H17A	109.5
C8—C7—C6	114.67 (18)	O3—C17—H17B	109.5
C16—C8—C9	119.21 (19)	H17A—C17—H17B	109.5
C16—C8—C7	120.17 (18)	O3—C17—H17C	109.5
C9—C8—C7	120.62 (18)	H17A—C17—H17C	109.5
C10—C9—C8	121.3 (2)	H17B—C17—H17C	109.5
C10—C9—H9	119.3		
			
C16—O1—C1—C2	−178.86 (17)	C9—C10—C11—C15	−1.3 (3)
C16—O1—C1—C6	1.1 (3)	C9—C10—C11—C12	179.5 (2)
O1—C1—C2—C3	179.87 (19)	C15—C11—C12—C13	−1.1 (3)
C6—C1—C2—C3	−0.1 (3)	C10—C11—C12—C13	178.0 (2)
C1—C2—C3—C4	0.3 (3)	C11—C12—C13—C14	−0.4 (4)
C2—C3—C4—C5	−0.2 (4)	C15—N1—C14—C13	−0.6 (3)
C3—C4—C5—C6	−0.1 (4)	C12—C13—C14—N1	1.4 (4)
O1—C1—C6—C5	179.83 (19)	C14—N1—C15—C11	−1.0 (3)
C2—C1—C6—C5	−0.2 (3)	C14—N1—C15—C16	178.70 (19)
O1—C1—C6—C7	−1.3 (3)	C12—C11—C15—N1	1.9 (3)
C2—C1—C6—C7	178.69 (19)	C10—C11—C15—N1	−177.29 (19)
C4—C5—C6—C1	0.3 (3)	C12—C11—C15—C16	−177.82 (18)
C4—C5—C6—C7	−178.6 (2)	C10—C11—C15—C16	3.0 (3)
C1—C6—C7—O2	179.7 (2)	C1—O1—C16—C8	0.2 (3)
C5—C6—C7—O2	−1.5 (3)	C1—O1—C16—C15	−179.36 (16)
C1—C6—C7—C8	0.2 (3)	C9—C8—C16—O1	179.58 (18)
C5—C6—C7—C8	179.03 (19)	C7—C8—C16—O1	−1.2 (3)
O2—C7—C8—C16	−178.4 (2)	C9—C8—C16—C15	−0.9 (3)
C6—C7—C8—C16	1.0 (3)	C7—C8—C16—C15	178.25 (19)
O2—C7—C8—C9	0.7 (3)	N1—C15—C16—O1	−2.1 (3)
C6—C7—C8—C9	−179.84 (18)	C11—C15—C16—O1	177.68 (17)
C16—C8—C9—C10	2.7 (3)	N1—C15—C16—C8	178.38 (18)
C7—C8—C9—C10	−176.5 (2)	C11—C15—C16—C8	−1.9 (3)
C8—C9—C10—C11	−1.6 (3)		

### Hydrogen-bond geometry (Å, °)

**Table:**

*D*—H···*A*	*D*—H	H···*A*	*D*···*A*	*D*—H···*A*
O3—H3···N1	0.82	2.07	2.852 (2)	160

## References

[bb1] Barbour, L. J. (2001). *J. Supramol. Chem.* **1**, 189–191.

[bb2] Bruker (2004). *APEX2* and *SAINT* Bruker AXS inc., Madison, Wisconsin, USA.

[bb3] Chen, Q., Qin, J.-K., Zeng, M.-H. & Ng, S. W. (2007). *Acta Cryst.* E**63**, o453–o454.

[bb4] Fujiwara, H. & Okabayashi, I. (1994). *Heterocycles*, **38**, 541–550.

[bb5] Sheldrick, G. M. (2008). *Acta Cryst.* A**64**, 112–122. 10.1107/S0108767307043930 18156677

[bb6] Westrip, S. P. (2009). *publCIF* In preparation.

